# Identification of alkaloids and related intermediates of *Dendrobium officinale* by solid-phase extraction coupled with high-performance liquid chromatography tandem mass spectrometry

**DOI:** 10.3389/fpls.2022.952051

**Published:** 2022-08-04

**Authors:** Cheng Song, Yunpeng Zhang, Muhammad Aamir Manzoor, Guohui Li

**Affiliations:** ^1^College of Biological and Pharmaceutical Engineering, West Anhui University, Lu’an, China; ^2^Shanghai Key Laboratory of Regulatory Biology, School of Life Sciences, East China Normal University, Shanghai, China; ^3^College of Life Sciences, Anhui Agricultural University, Hefei, China

**Keywords:** hormone elicitor, liquid chromatography-mass spectrometry, alkaloid, *Dendrobium*, solid-phase extraction (SPE)

## Abstract

Jasmonate (JA) signaling plays a pivotal role in plant stress responses and secondary metabolism. Many studies have demonstrated that JA effectively induce the expressions of alkaloid biosynthetic genes in various plants, which rendered to the accumulation of alkaloid to counteract stresses. Despite the multiple roles of JA in the regulation of plant growth and different stresses, less studied involved in the regulatory role of JA in *Dendrobium officinale* alkaloids. A strategy for the rapid identification of alkaloid and the intermediates of *D. officinale* was established based on a solid-phase extraction coupled with high-performance liquid chromatography tandem mass spectrometry method. By using SPE-LC-MS/MS method, the potential compounds were tentatively identified by aligning the accurate molecular weight with the METLIN and Dictionary of Natural Products databases. The chemical structures and main characteristic fragments of the potential compounds were further confirmed by retrieving the multistage mass spectra from the MassBank and METLIN databases. The Mass Frontier software was used to speculate the fragmentation pathway of the identified compounds. Seven alkaloids were separated and identified from *D. officinale*, which were mainly classified into five types (tropane alkaloids, tetrahydroisoquinoline alkaloids, quinolizidine alkaloids, piperidine alkaloids, and spermidine alkaloids). Besides the alkaloids, forty-nine chemical substances, including guanidines, nucleotides, dipeptides, sphingolipids and nitrogen-containing glucosides, were concurrently identified. These findings gives the composition of chemicals currently found in *D. officinale*, which could provide the scientific method for the identification of alkaloids in other *Dendrobium* plants.

## Introduction

Traditional Chinese medicine (TCM) involves complex chemical constituents. The successful isolation of these natural compounds is required for pharmacological and pharmacodynamic investigations. Moreover, A separation and identification method with strong specificity, high efficiency, and few interference is urgently needed. Compared with high-performance liquid chromatography (HPLC), conventional detection method such as thin-layer chromatography (TLC) provide low accuracy and sensitivity in the qualitative and quantitative analysis of small molecules (Patel et al., 2008; Pan et al., 2015). Solid-phase extraction (SPE) was typically used for the complicated specimens by reducing matrix interference and raising sensitivity (Bucar et al., 2013). Combined with the HPLC method, SPE-HPLC technology solves problems such as interference and co-elution of target compounds by other impurities. Orbitrap mass spectrometry is one kind of high-resolution mass analyzer containing multistage fragment monitoring, a selective fragmentation mode, and a personalized data acquisition method, which is widely applied in pharmacokinetic analysis, the food industry, environmental monitoring, and proteomics (Li et al., 2013; Qiu et al., 2015; Quifer-Rada et al., 2015).

*Dendrobium officinale*, as a type of perennial herb, is attached to the *Dendrobium* genus of Orchidaceae (Song et al., 2021). As a valuable TCM, *D. officinale* contains many different types of active ingredients, including polysaccharides, alkaloids, bibenzyls, phenanthrenes, sesquiterpenoids, fluorenones, flavonoids, steroids, etc. The earliest studies on the *Dendrobium* alkaloids were derived from *D. nobile* (Xu et al., 2010). Some alkaloids like dendrobine and nobiline had exhibited good pharmacological and pharmacodynamic activity (Ng et al., 2012). For the decades, dozens of alkaloids have been subsequently identified from *D. anosmum*, *D. chrysanthum*, *D. crepidatum*, *D. findlayanum*, *D. friedricksianum*, *D. hilderbrandii*, *D. loddigessi*, *D. lohohense*, *D. moniliforme*, *D. parishii*, *D. pierardii*, *D. primulimun*, *D. snowflake* and *D. wardianum* (Morita et al., 2000; Liu et al., 2007; Xu et al., 2013). The alkaloids identified from Dendrobiun plants could be divided into five categories based on their chemical structures, including sesquiterpenoid alkaloids, indolizidine alkaloids, pyrrolidine alkaloids, phthalide alkaloids, and imidazole alkaloids (Song et al., 2022b). Although different types of alkaloids have been isolated from many *Dendrobium spp.*, the composition and biosynthesis of the alkaloids in *D. officinale* remain unsolved issues.

Jasmonates have a crucial function in the stress responses and biosynthesis of secondary metabolites throughout plant development. In *D. officinale*, the key genes related to MVA and MEP pathway and some post-modified enzymes were significantly up-regulated by MeJA treatment (Chen et al., 2019). High-yielding production of alkaloids from *D. officinale* was obtained with the combination of tryptophan, secologanin, and MeJA treatment (Jiao et al., 2018; Song et al., 2020, Song et al., 2022a). The genome and transcriptome sequencing of *D. officinale* implied that a biosynthetic pathway oriented to the terpenoid indole alkaloids (TIAs) likely exists (Guo et al., 2013; Yan et al., 2015). Recently, high-throughput sequencing revealed the identification of a total of 56 genes, including 25 key enzymes potentially involved in the biosynthesis of TIAs, tropine alkaloids, and isoquinoline alkaloids (Niu et al., 2021). Many studies had demonstrated that *CrMYC2* and *ORCAs* transcription factors may play a positive role in elevating the TIAs levels in *Catharanthus roseus* (Memelink and Gantet, 2007). With the help of the transcriptome sequencing of *D. officinale*, 75 *bHLH* genes, and 66 *AP2/ERF* genes were concurrently identified in the DEGs, which suggested that those transcription factors could be involved in the regulation of secondary metabolism, especially in TIAs biosynthesis (Zhang et al., 2016; Song et al., 2022c).

In this study, a combination method for separating and detecting alkaloids and their precursors was established. The accurate mass of the components separated by HPLC were determined using an high resolution mass analyzer. The multistage fragmentation pathway of alkaloids was speculated based on a database-dependent retrieval method. The results would provide a reliable scientific reference and technical guidance for the identification of other *Dendrobium* alkaloids.

## Materials and methods

### Instruments and chemicals

The LTQ-Orbitrap XL mass spectrometer manufactured by Thermo Fisher Scientific (Bremen, Germany) was used for the mass spectrometry analysis of ionized substances. The Agilent 1260 instrument manufactured by Agilent Technologies (Waldbronn, Germany) was used for the chromatographic separation of methanol extract of *D. officinale*. The Xcalibur (*v.* 2.1) and Mass Frontier (*v.* 6.0) softwares were used for analyzing mass spectrometry and speculating of the fragmentation pathways of unmatched compounds.

Acetonitrile and formic acid as mobile phases were of HPLC grade. Other chemicals used in the experiment were domestically made analytical reagents. The standards reserpine and sarpagine were purchased from Aladdin (Shanghai, China) and BioBioPha (Kunming, China), respectively.

### Plant samples preparation and total alkaloids extraction

The *D. officinale* samples used in the experiment were collected from the tissue culture room of the Plant Cell Engineering Center at West Anhui University. The tissue-cultural seedlings were cultivated in MS medium without additional hormones. The optimal concentration of 100 M MeJA was then applied to *D. officinale* samples, which manifested in the accumulation of total alkaloids. The seedlings were firstly dried at 110°C for 15 min and kept in a constant weight at 60°C. The dried materials were ground into powders by a universal grinder. Using the ultrasonic extraction method, two gram of powder was placed in a round-bottom flask with 100 mL of methanol and extracted three times at room temperature. The bromocresol green colorimetric method was used for the determination of total alkaloids.

The extraction solution was centrifuged for 10 min at 6,000× *g* to get the supernatant, then evaporated to dryness in a rotary evaporator. Dilute sulfuric acid (5 mL) was added to dissolve the extraction, followed by filtering through a 0.22 μm membrane, before being pretreated using MCX cartridges (60 mg, 3 mL). The protocol was successively conducted by our previous method (Song et al., 2020).

### The LC-MS conditions and the interpretation of multistage mass spectrometry

The Waters Atlantis T3 reversed-phase column (150 mm × 4.6 mm, 3 μm) was used for the chromatographic separation. The mobile phases acetonitrile (A) added with 0.1% formic acid and aqueous phase (B) added with 0.1% formic acid were used for the experiment. Gradient elution process was set as follows: 0–2 min (0% A), 2–12 min (0–15% A), 12–22 min (15–35% A), 22–32 min (35–80% A), and 32–37 min (80–0% A). The UV wavelength was set at 280 nm, and the column temperature was set at 25°C. The volume flow for chromatographic separation was at 1.2 mL/min. To meet the conditions of mass spectrometry detection, a three-way splitter was connected with the mass analyzer at a 0.3 mL min^–1^ flow rate. The ion-source parameters were refereed to Kumar’s method and slightly modified (Kumar et al., 2016). The detailed parameters were as follows: sheath gas at 25 arb, auxiliary gas at 3 arb, spray voltage at 4 kV, the capillary temperature at 320°C, tube lens at 120 V, and capillary voltage at 30 V. The MS data were collected at 100 ≤ *m*/*z* ≤ 1000 in positive-ion mode.

At the stage of data acquisition, two data acquisition methods were used in the experiments. In one method, a high-resolution scan was conducted using the Orbitrap mass analyzer to acquire the MS data at a resolution at 30,000 FWHM and the MS^2^ data at 15,000 FWHM. A data-dependent MS^n^ scan was used for the analysis of the MS^2^ spectra generated from the most abundant ions of the MS spectra Kalli et al., 2013. In the other method, a high-resolution scan was conducted first to acquire the MS data at a resolution at 30,000 FWHM, then the LTQ dynode was used to scan the MS^2^ and MS^3^ spectra. The dynamic exclusion function was used to reduce repeat scans, and the repeat count was 2. The exclusion duration was 20 s, and the exclusion mass width was 3 *m*/*z*. The optimized collision-induced dissociation (CID) energy was set at 35%. The minimum signal threshold was 500, and the isolation width of precursor ions was 2 *m*/*z.* Sarpagine and reserpine, two standards of TIAs, were, respectively injected into the mass analyzer *via* a syringe pump at a flow rate of 6 μL/min to complete the direct infusion analysis. Using Xcalibur software, the putative molecular formulae of those adducted molecular ions were recalculated and obtained based on the accurate molecular weight. Most compounds were tentatively identified by matching their mass spectra to METLIN, Mass Bank, Dictionary of Natural Products, and Chemspider databases. For the compounds without multiple mass spectra, Mass Frontier software was helped to speculate and deduce the fragmentation pathway. The raw datasets were uploaded to Dryad in curation.^1^

## Results and discussion

### Determination of biomass and alkaloid contents under MeJA treatment

**FIGURE 1:**
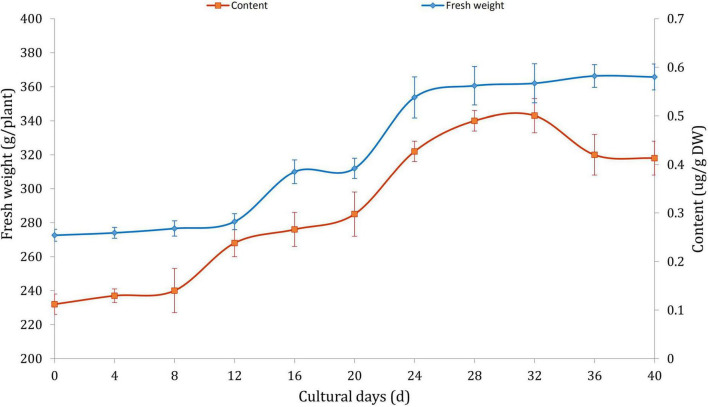
The fresh weight and alkaloid contents of *Dendrobium officinale* treated with 100 μM MeJA.

The effects of MeJA on the growth and alkaloid accumulation of *D. officinale* was investigated. The results indicated that MeJA delayed the growth rate of *D. officinale* and initiated the biosynthesis of a large number of alkaloids in logarithmic growth phase (Figure 1). The highest content of alkaloids climaxed to 343 μg/g when cultured on the 32 days. After that, the fresh weight tended to be stable, and the alkaloid content began to fall gradually. The results suggested that JA could continuously promote the biosynthesis of alkaloids during the growth of *D. officinale*. The growth trend and accumulating pattern were consistent with the previous studies (Jiao et al., 2018; Wang et al., 2021).

### Mass spectra interpretation of the main compounds in *Dendrobium officinale*

Firstly, *D. officinale* samples were extracted by methanol and dissolved with diluted sulfuric acid. The MCX cartridges were used for the purification of total alkaloids from the acidic aqueous extraction. To optimize the conditions of mass spectrometry, sarpagine and reserpine, two important terpenoid indole alkaloids, were applied as internal standards to deduce the fragmentation pathway of multi-stage MS. The suitable CID energy was achieved by the direct infusion experiment. Generally, the strongest fragment ions and the smallest number of fragments were observed at 30–35% collision energy, whereas the abundances of the fragments decreased substantially at 45% or greater collision energy. In this experiment, sarpagine and reserpine achieved sufficient fragments both in quantity and intensity at 40% collision energy. High-resolution mass analysis was conducted by a data-dependent MS^n^ scan method from the workstation to produce abundant fragmentation spectra of the product ions. Due to the high resolution, the scan rate of a single spectrum was reduced, and the analytical time of the entire sample was extended. An appropriate exclusion duration time was required for the dynamic exclusion function to avoid repeat scans of target ions and thereby shorten the analysis time of the samples. Dynamic exclusion could also be used to obtain the fragment information of the co-eluting small peaks, which allowed compounds with low ionized potentials to be detected (Pan et al., 2015). The total ion current chromatogram of the methanol extract spiked with standards from *D. officinale* is presented, as obtained in the positive-ion mode under the optimized mass parameters and analytical conditions (Supplementary Figure 1A). Generally, [M + H]^+^ or [M]^+^ was achieved through the soft ionization in the positive mode. The molecular-ion peaks of sarpagine and reserpine were separately extracted at *m/z* 311.1738 and 609.2772. The retention time of sarpagine and reserpine were at 12.95 min and 25.41 min, respectively (Supplementary Figures 1B,C). The results indicated that the established method for the mass spectrometry was suitable for the separation and characterization of alkaloids.

Using a data-dependent MS^n^ scan method, the MS spectra of two standards were obtained from the Orbitrap analyzer, and the MS^n^ spectra were obtained from the LTQ. It was indicated that the π bonds on the indole groups of sarpagine and reserpine were easily dissociated to produce [M + H]^+^ peaks and produced massive hydrogen rearrangement. The ionization and electron transfer were observed on the position of the group cleavage because of the induced dissociation effect of the high-energy collisions. The putative fragmentation modes of sarpagine and reserpine are presented in Supplementary Figures 2, 3. The characteristic fragments with *m/z* 146 and 174 from the indole group were produced by the cleavage of sarpagine and reserpine, respectively. The characteristic ion *m/z* 195 cleaved on the ester bond was obtained to produce the 3,4,5-trimethoxybenzaldehyde group from the aglycone. Finally, a total of forty-nine nitrogen-containing compounds, including seven alkaloids, were separated and tentatively identified from *D. officinale* (Table 1).

**TABLE 1:** Identification of main chemical compounds from *Dendrobium officinale.*

*t*_R_ (min)	[M + H]^+^	Formula	Error (mmu)	Fragment ions	Identification
2.64/3.19	118.0855	C_5_H_12_O_2_N	−0.795	MS^2^[118]:72 MS^3^[118→72]:55	L-Valine
5.89/6.24	132.1010	C_6_H_14_O_2_N	−0.905	MS^2^[132]:86 MS^3^[132→86]:69;58	L-Isoleucine
3.81/7.73	136.0609	C_4_H_10_O_4_N	−0.832	MS^2^[136]:119;118;108;107;100;91;57	4-Hydroxy-L-threonine
5.00/6.42	139.0494	C_6_H_7_O_2_N_2_	−0.784	MS^2^[139]: 121;111;95	Urocanic acid
2.81/3.02	142.1218	C_8_H_16_ON	−0.841	MS^2^[142]: 124;114;98;82;70;55	Tropine or its isomers
5.14/5.65	144.1011	C_7_H_14_O_2_N	−0.835	MS^2^[144]:126;86;84 MS^3^[144→84]:56	2,3-Dihydroxynortropane or its isomers
3.71	146.0914	C_5_H_12_O_2_N_3_	−0.973	MS^2^[146]: 128;104;101;87;86;60 MS^3^[146→128]:111;86;84	4-Guanidinobutanoic acid
1.21	147.1120	C_6_H_15_O_2_N_2_	−0.854	MS^2^[147]:130; 129;84 MS^3^[147→130]:84	L-Lysine
4.06	152.0559	C_5_H_6_ON_5_	−0.806	MS^2^[152]:136;135;134;124;110;109;90 MS^3^[152→135]:107;92;77	2-Hydroxyadenine
5.56	154.0967	C_7_H_12_ON_3_	−0.839	MS^2^[154]:136;112;94;70 MS^3^[154→112]:95;94;84;70;69;67	Cyclocimipronidine
1.29	156.0759	C_6_H_10_O_2_N_3_	−0.873	MS^2^[156]: 110; 95 MS^3^[156→110]: 93; 83	L-Histidine
6.42	160.1070	C_6_H_14_O_2_N_3_	−1.023	MS^2^[160]:143;142;125;104;87;86;74 MS^3^[160→104]:87;86	*N-tert*-Butyloxycarbonyl guanidine
8.88	166.0853	C_9_H_12_O_2_N	−1.005	MS^2^[166]:120 MS^3^[166→120]:120;103;93;91	L-Phenylalanine
1.36	175.1178	C_6_H_15_O_2_N_4_	−1.182	MS^2^[175]: 158;140;130;116;112;70;60	L-Arginine
12.47	180.1007	C_10_H_14_O_2_N	−1.225	MS^2^[180]:163;120 MS^3^[180→120]:120;103;93	β-Phenyl-γ-aminobutyric acid
6.79	180.1008	C_10_H_14_O_2_N	−1.075	MS^2^[180]:163;145;137 MS^3^[180→163]:145;117	L-Homophenylalanine
7.00	182.0801	C_9_H_12_O_3_N	−1.06	MS^2^[182]:165;147;136	L-Tyrosine
1.60	189.1336	C_7_H_17_O_2_N_4_	−0.982	MS^2^[189]:172;171;158;144;133;116;115;74;70 MS^3^[189→172]:141;126;116;115	*N^g^*-Methyl-L-arginine
7.34/7.65/8.73/9.43	203.1379	C_9_H_19_O_3_N_2_	−1.199	MS^2^[203]:185;157;132;86 MS^3^[203→132]:86;69	Alanyl-isoleucine or its isomers
11.86	205.0960	C_11_H_13_O_2_N_2_	−1.144	MS^2^[205]:188 MS^3^[205→188]:170;146;144	L-Tryptophan
4.09	217.1285	C_8_H_17_O_3_N_4_	−1.037	MS^2^[217]:200;199;175;158;157;139;115;113;70 MS^3^[217→200]:183;158;157;139;115; 113;70	*N*^α^ -Acetyl-L-arginine
8.04	217.1534	C_10_H_21_O_3_N_2_	−1.239	MS^2^[217]:199;171;118;72	Valyl-valine
27.88	225.1945	C_13_H_25_ON_2_	−1.59	MS^2^[225]:207;143;100;83; MS^3^[225→100]:83	Anapheline
26.46	230.2462	C_14_H_32_ON	−1.641	MS^2^ [230]:212;185; MS^3^[230→212]: 194; 169;156;125;111;97;85;71;69	2-Amino-3-tetradecanol
9.98/10.6/11.48	231.1690	C_11_H_23_O_3_N_2_	−1.309	MS^2^[231]:213;185;132;72 MS^3^[231→72]:55	Valyl-leucine or its isomers
14.93	237.1219	C_12_H_17_O_3_N_2_	−1.469	MS^2^[237]:219;205;180;177;175;148 MS^3^[237→175]:160;158;148;134;132;94	*N*^δ^ -Benzoylornithine
9.77	237.1219	C_12_H_17_O_3_N_2_	−1.469	MS^2^[237]:219;120 MS^3^[237→120]: 120;103;93;91	Carbetamide
4.25	244.0913	C_9_H_14_O_5_N_3_	−1.487	MS^2^[244]:112 MS^3^[244→112]:112;95	Cytidine
12.55/13.24/13.59/14.35	245.1844	C_12_H_25_O_3_N_2_	−1.599	MS^2^[245]:227;199;132;86 MS^3^[245→86]:69	Leucyl-isoleucine or its isomers
10.37	253.1280	C_11_H_17_O_3_N_4_	−1.557	MS^2^[235]:236;235;225;208;193;192; 191;181;165;164;147;112 MS^3^[235→236]:218;209;193; 192;191;175;165; 147;121;70	Prolyl-histidine
11.41/12.73	265.1531	C_14_H_21_O_3_N_2_	−1.529	MS^2^[265]:248;206;177;152;114;89 MS^3^[265→248]:177;145	Phenylalanyl-valine or its isomers
7.73	268.1025	C_10_H_14_O_4_N_5_	−1.58	MS^2^[268]:136 MS^3^[268→136]:136;119;94;82	Adenosine
26.34	274.2720	C_16_H_36_O_2_N	−2.026	MS^2^[274]: 256;230;102;88; MS^3^[274→256]: 238;212;102;88	2-Amino-1,3-hexadecanediol
14.93/15.67/16.20/16.48	279.1685	C_15_H_23_O_3_N_2_	−1.739	MS^2^[279]:261;233;205;149;132;120; MS^3^[279→120]:103;93;91	Isoleucyl-phenylalanine or its isomers
8.73	282.1181	C_11_H_16_O_4_N_5_	−1.52	MS^2^[282]:136 MS^3^[282→136]:136;119;94	1-Methyladenosine
8.20	284.0973	C_10_H_14_O_5_N_5_	−1.685	MS^2^[284]:152 MS^3^[284→152]:152;135;110;109	Guanosine
26.59	290.2670	C_16_H_36_O_3_N	−1.97	MS^2^[290]:272;242;122; MS^3^[290→242]:88	2-Amino-1,3,4-hexadecanetriol
4.64	291.1286	C_10_H_19_O_6_N_4_	−1.311	MS^2^[291]:175 MS^3^[291→175]: 158;157; 130;116;112;70;60	*N*^2^-(3-Hydroxysuccinoyl)arginine
11.53/12.0/12.37/ 13.34	295.1635	C_15_H_23_O_4_N_2_	−1.724	MS^2^[295]:277;249;182;165;86; MS^3^[295→277]: 259;231;166;120	Isoleucyl-tyrosine or its isomers
11.66	297.1541	C_18_H_21_O_2_N_2_	−5.614	MS^2^[297]:265;248 MS^3^[297→265]: 248;221;204;187;176; 161;112	Alamaridine
7.52	314.0901	C_13_H_16_O_8_N	3.047	MS^2^[314]:296;278;136;97 MS^3^[314→136]:136;119;94	4-*O*-β-D-Glucopyranoside- 2,4-benzoxazolediol
26.46	318.2982	C_18_H_40_O_3_N	−2.111	MS^2^[318]:300;256; MS^3^[318→256] 228;212;102;88	2-Amino-1,3,4-octadecanetriol
6.87	332.1324	C_14_H_22_O_8_N	−1.643	MS^2^[332]: 314;233;170;152;136;108	5′-*O*-beta-D-Glucosylpyridoxine
1.48	337.1701	C_12_H_25_O_7_N_4_	−1.706	MS^2^[337]:319;301;283;260;257;217; 209;175;173;158;112 MS^3^[337→319]: 301; 283; 275;260;257; 239;209; 175; 158;112	*N*^2^-Fructopyranosylarginine
19.43	348.1784	C_19_H_26_O_5_N	−2.139	MS^2^ [348]: 207;175;142; 122; MS^3^[348→142]:142;124;122;96;70	1,2-Dihydro-*O*-methyltazettine
9.69	418.1685	C_18_H_28_O_10_N	−2.282	MS^2^[418]: 400;286;238;148 MS^3^[418→238]:220;208;202; 190; 174;172;164; 148;146;134;118;108;106	Passicapsin
16.78	438.2359	C_25_H_32_O_4_N_3_	−2.863	MS^2^[438]:421;292;275;218;204;147; MS^3^[438→204]:147	Meefarnine B
9.16/9.51	454.1685	C_21_H_28_O_10_N	−2.282	MS^2^[454]:322;160 MS^3^[454→322]:304;160;142	*O*-(tri-*O*-Acetyl-α-L-rhamnopyranoside)-(4-hydroxybenzyl) methylcarbamic acid
16.05	454.2301	C_26_H_32_NO_6_	7.686	MS^2^[454]: 437;308;292;275;234;220;204;163;147	Methyllagerine *N*-oxide

### Identification of the alkaloids in *Dendrobium officinale*

The small molecular compounds previously identified from *D. officinale* mainly included stilbenoids (bibenzyl and phenanthrene), phenols, lignans, nucleotides, lactones, flavonoids, steroids, and fatty acids (Xu et al., 2013). Based on the high resolution and sensitivity of LTQ-Orbitrap for trace substances, coupled with the high selectivity of MCX for medium-strength alkaline compounds, seven alkaloids identified from the methanol extract of *D. officinale* were divided into five classes based on the chemical structure, including tropane alkaloids, tetrahydroisoquinoline alkaloids, quinolizidine alkaloids, piperidine alkaloids, and spermidine alkaloids. The special fragmentation modes of different alkaloids in the ESI source were discussed below.

**FIGURE 2:**
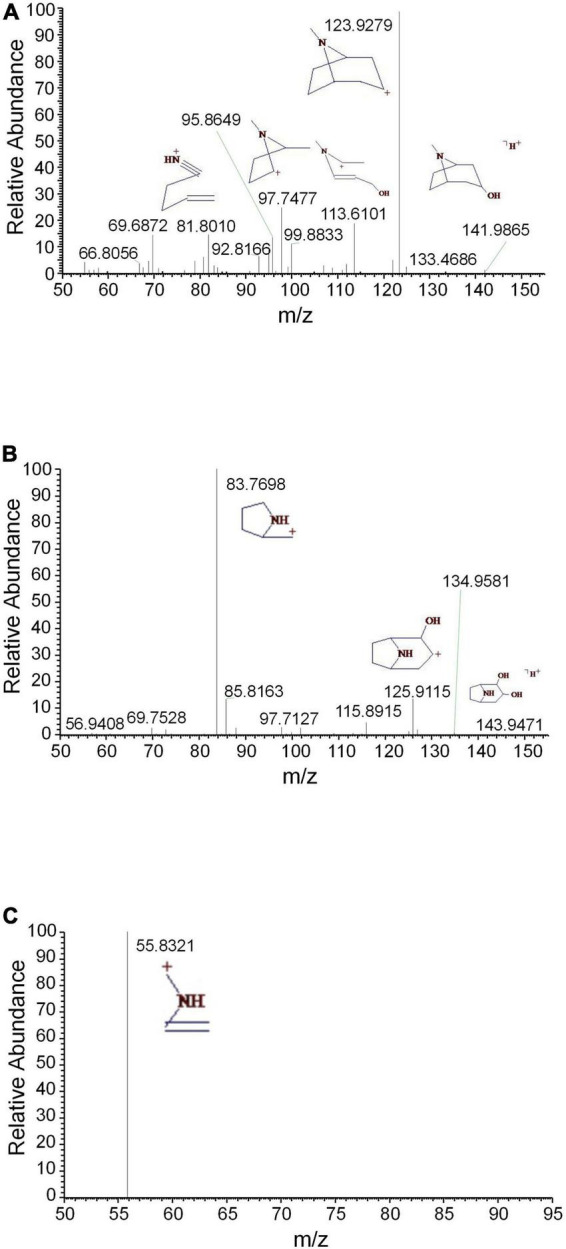
The mass spectra of tropine and 2,3-dihydroxynortropane. **(A)** The MS^2^ spectrum of tropine, **(B)** the MS^2^ spectrum of 2,3-dihydroxynortropane or an isomer, and **(C)** the MS^3^ spectrum of 2,3-dihydroxynortropane or an isomer.

**FIGURE 3:**
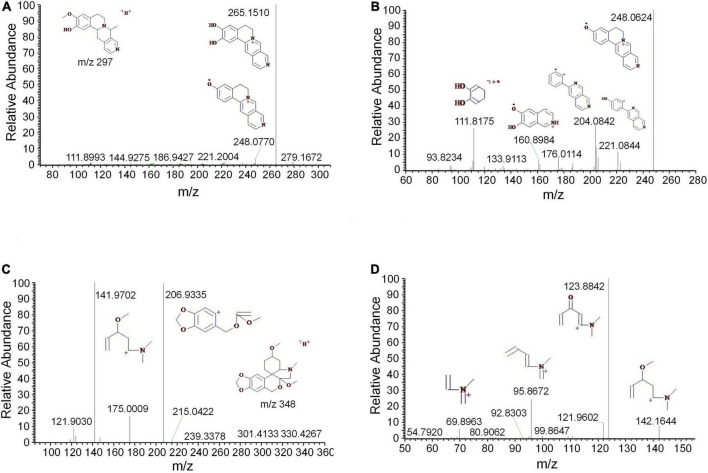
The mass spectra of alamaridine and 1,2-dihydro-*O*-methyltazettine. **(A)** The MS^2^ spectrum of alamaridine, **(B)** the MS^3^ spectrum of alamaridine, **(C)** the MS^2^ spectrum of 1,2-dihydro-*O*-methyltazettine, and **(D)** the MS^3^ spectrum of 1,2-dihydro-*O*-methyltazettine.

Tropane alkaloids consist of pyridine conjugated with piperidine. The DART-MS in-source CID indicated that the characteristic ions of tropine consisted of *m/z* 158, 142, 124, 93, and 67 (Lesiak et al., 2015). 2,3-Dihydroxynortropane was a kind of nortropane-type alkaloid that lacked an N-methyl group (Asano et al., 2001). Notably, the hydroxyl groups presented on its C-2 and C-3 included both the α- and β-configurations (Kusano et al., 2002). In this study, a strong [M + H-⋅OH]^+^ ion was observed under the set CID conditions. In addition to the characteristic ions 93 and 67, the stronger fragments 113, 98, and 82 were observed (Figure 2A). The base peak of 2,3-dihydroxy- nortropane was the 2-methylpyrrole ion, which was further fragmented to get *m/z* 56. Finally, the compound was identified as 2,3-dihydroxynortropane or its isomers (Figures 2B,C). Two tetrahydroisoquinoline alkaloids were identified as alamaridine and 1,2-dihydro-*O*-methyltazettine. The characteristic ions of tetrahydroisoquinoline group in alamaridine were *m/z* 177, 176, and 162 in the EI source, whereas the methyl group on C-5 was easier to lost compared to that on C-10 Bhattacharjya et al., 1986. The MS^2^ and MS^3^ spectra of alamaridine are displayed, respectively (Figures 3A,B). These spectra suggested that the methyl groups on C-5 and C-10 were lost to produce the base peaks [M + H-2CH_3_-3H]^+^, which further lacked a hydroxyl group to generate *m/z* 248 in the MS^3^ spectrum. The C_2_H_3_ and ⋅OH groups were successively cleaved to generate *m/z* 221 and 204 on ring B, and *m/z* 176 and 161 on ring C. 1,2-Dihydro-*O*-methyltazettine, also known as ungvedine, is a tazettine-type alkaloid (Kadyrov et al., 1979). The main fragments *m/z* 207 and 142 were generated by 1,2-dihydro-*O*-methyltazettine with equivalent intensity (Figure 3C). The *m/z* 142 further lacked the methyl group and simultaneously formed two double bonds to generate the base peak *m/z* 124 (Figure 3D). Previous studies had shown that the fragmentation pattern of tazettine-type alkaloid, of which C_3_H_7_N group was easily lost to produce the characteristic ion [M + H-57]^+^ (Zhang et al., 2009). 1,2-dihydro-*O*- methyltazettine was fragmented by RDA cleavage at both C-6, N-5 and C-4b, C-6a (Figure 3C). 1,2-Dihydro-*O*-methyltazettine cleaved at C-4b, C-12a but not on C-3, C-4, and thus did not produce [M + H-C_3_H_7_N]^+^. There was a coordination bond between nitrogen and oxygen presented in the quinolizidine alkaloid methyllagerine *N*-oxide. It was indicated that this bond easily led to the generation of the fragment [M-O]^+^ in the EI source (Lee et al., 2011).

**FIGURE 4:**
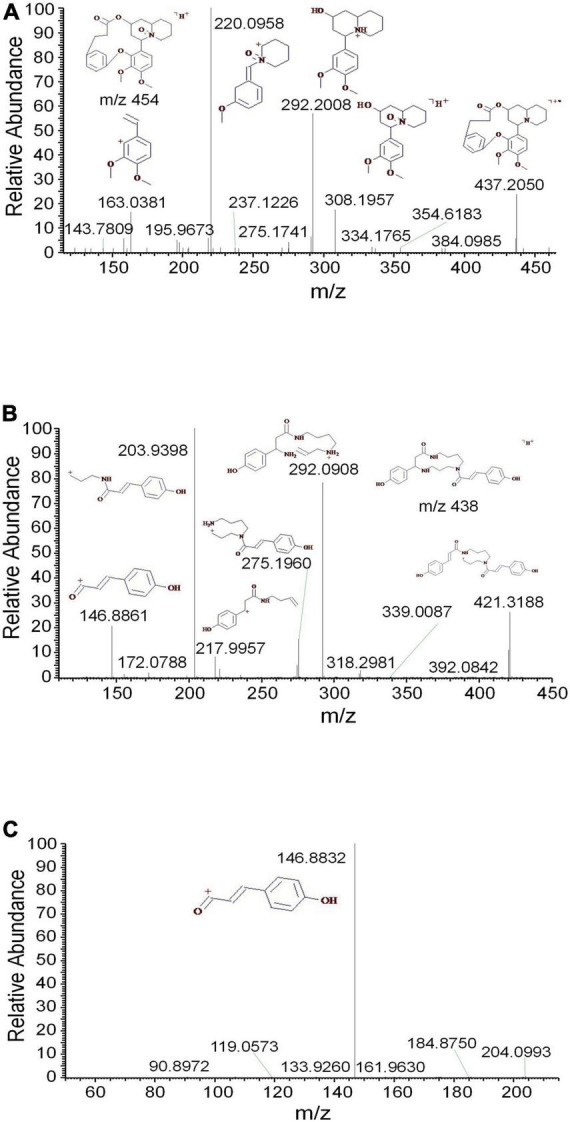
The mass spectra of methyllagerine *N*-oxide and meefarnine B. **(A)** The MS^2^ spectrum of methyllagerine *N*-oxide, **(B)** the MS^2^ spectrum of meefarnine B, and **(C)** the MS^3^ spectrum of meefarnine B.

**FIGURE 5:**
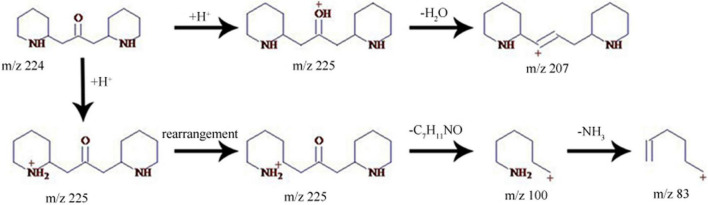
The putative fragmentation pathway of anapheline.

Additionally, the oxygen was easily lost as well to produce the fragment *m/z* 437 in the ESI source (Figure 4A). The characteristic ions *m/z* 308 and 292 were generated by the cleavage of C-11 and C-22. According to the different cleavage positions on ring B, the characteristic ions *m/z* 220 and 163 were also generated. Meefarnine B was a spermidine alkaloid containing a polyamine heterocyclic ring. These alkaloids are generally considered to be synthesized by putrescine, which is produced by the degradation of L-arginine or L-ornithine (da Silva et al., 2015). The MS^2^ and MS^3^ spectra of meefarnine B indicated that the NH_3_ group on N-5 was cleaved to produce the *m/z* 421 due to the collision dissociation effect (Figures 4B,C). The fragment 292 was generated by the cleavage of the side chain on N-9 and was further cleaved to produce *m/z* 218. Besides, the fragment 275 was generated by the cleavage of the *m/z* 421 on N-1 and C-2. The fragment 204 was generated by the cleavage of the *m/z* 275 on N-9 and C-10, which further produced the characteristic ion 147. In this study, anapheline was tentatively identified from *D. officinale*. The retention time of anapheline was 27.88 min, and the molecular weight of the molecular ion was 225.1945, whichsuggested that its molecular formula was C_13_H_25_ON_2_ (Supplementary Figure 4). Anapheline easily lost H_2_O to produce the fragment *m/z* 207 and the piperidine ion (*m/z* 100) during the collision-induced dissociation. The NH_3_ group was lost to produce the *m/z* 83 in the MS^2^ spectrum. Based on the MS^2^ and MS^3^ spectra, a presumed fragmentation pathway of anapheline was proposed (Figure 5).

### Identification of the guanidines in *Dendrobium officinale*

**FIGURE 6:**
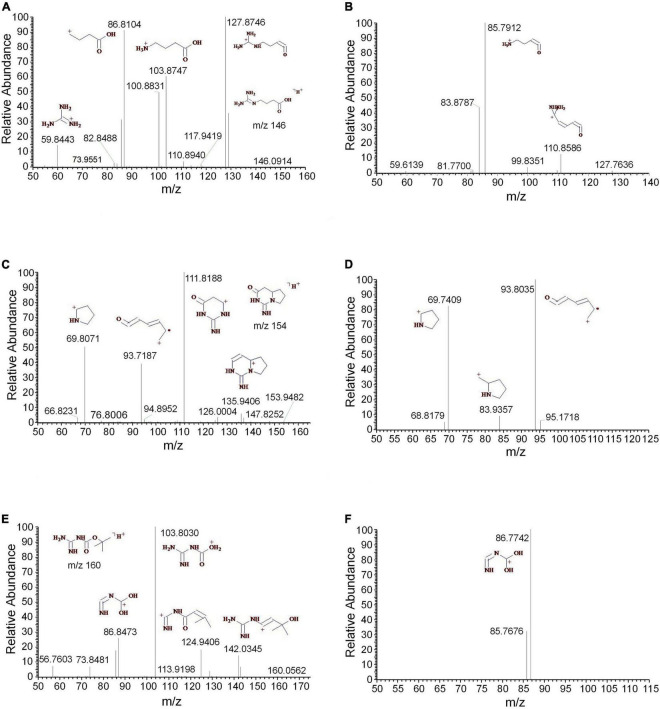
The mass spectra of the guanidines. **(A)** The MS^2^ spectrum of 4-guanidinobutanoic acid, **(B)** the MS^3^ spectrum of 4-guanidinobutanoic acid, **(C)** the MS^2^ spectrum of cyclocimipronidine, **(D)** the MS^3^ spectrum of cyclocimipronidine, **(E)** the MS^2^ spectrum of *N*-*tert*-butyloxycarbonyl guanidine, and **(F)** the MS^3^ spectrum of *N*-*tert*-butyloxycarbonyl guanidine.

Three guanidine compounds were separated and determined from the methanol extract of *D. officinale* for the first time. The MS^2^ and MS^3^ spectra of 4-guanidinobutanoic acid were displayed, respectively (Figures 6A,B). The mass spectra indicated that 4-guanidinobutanoic acid easily lost H_2_O to form the *m/z* 128 and produced *m/z* 104, 87 and 60. The *m/z* 128 was further cleaved to produce the *m/z* 111 and 86. These results were consistent with those reported by the previous study (Nikolić et al., 2012). Cyclocimipronidine is a cyclic guanidine compound separated from *Cimicifuga racemosa*. Some studies showed that the characteristic ions of cyclocimipronidine, a guanidine alkaloid in black cohosh, were the *m/z* 112, 95, 94, 70, and 67 (Gödecke et al., 2009). In addition to the aforementioned characteristic ions, cyclocimipronidine easily lost H_2_O to generate the fragments *m/z* 136 and 84 (Figures 6C,D). The MS^2^ and MS^3^ spectra of *N*-*tert*-butyloxycarbonyl guanidine were displayed in Figures 6E,F, respectively. It was indicated that the characteristic ions were the *m/z* 142, 125, 104, and 87.

### Identification of the nucleotides and derivatives in *Dendrobium officinale*

Nucleotides were composed of purine or pyrimidine linked with ribose. Adenosine, uridine and guanosine had been separated from *D. officinale*. In this study, five nucleotide derivatives were tentatively identified. 2-Hydroxyadenine, as an adenine derivative, easily lost an NH_3_ group to produce the characteristic ions 135 and 110 (Supplementary Figure 5A). The fragments 107, 92, and 77 were generated in the MS^3^ spectrum (Supplementary Figure 5B). Besides 2-hydroxyadenine, another four nucleotide derivatives were cocurrently identified. Their MS^2^ and MS^3^ spectra indicated that the loss of aglycones caused the neutral loss of *m/z* 136 (Supplementary Figures 5C–J). Comparison with the mass spectra of the METLIN database^2^ resulted in the identification of adenosine, guanosine, 1-methyladenosine, and cytidine.

### Identification of the sphingolipids in *Dendrobium officinale*

Sphingolipid is a structurally complex lipid containing a long-chain sphingosine skeleton, which forms the plasma and vacuole membranes of plant cells. Four phytosphingosines were identified from *D. officinale*. Because sphingosine contained some hydroxyl groups, the characteristic ion [M + H-H_2_O]^+^ was observed during the dissociation (Supplementary Figures 6A–H). By retrieving the Dictionary of Natural Products Database,^3^ four sphingolipids, including 2-amino-1,3-hexadecanediol, 2-amino-3-tetradecanol, 2-amino-1,3,4-oct adecanetriol and 2-amino-1,3,4-hexadecanetriol, were identified in the experiment.

### Identification of the amino acids and their derivatives in *Dendrobium officinale*

Eight amino acids—L-lysine, L-histidine, L-arginine, L-valine, L-isoleucine, L-tyrosine, L-phenylalanine, and L-tryptophan – were identified from *D. officinale* based on accurate molecular weights and the multistage mass spectra of standards from the MassBank database^4^ (Table 1). 4-Hydroxy-l-threonine and urocanic acid were made from l-threonine and l-histidine. They easily lost H_2_O to make the base peaks m/z 118 and 121, respectively. The putative fragment ions generated during their cleavage were displayed in Supplementary Figures 7A,B, respectively.

In addition to the derivatives of L-threonine and L-histidine, four additional arginine derivatives were identified in this study. *N*^2^-Fructopyranosylarginine was an arginine monoglycoside and was considered as a byproduct generated by a Maillard reaction between reducing sugars (fructose or glucose) and arginine upon heating (Suzuki et al., 2004). Because fructose contained several hydroxyl groups, H_2_O was easily lost to produce the characteristic ions *m/z* 319, 301, and 283. The fragments *m/z* 257 and 239 were produced by the cleavage of the guanidino group on the glycoside. N^2^-Fructopyranosylarginine was also cleaved to generate the characteristic ion *m*/*z* 175 on the N-glucosidic band and further produced the aglycone *m/z* 158 *via* the neutral loss of fructose (Supplementary Figures 7C,D). The NH_3_ group of *N*-methylarginine was lost to produce the base peak *m/z* 172, as a result of the dissociation. According to the different cleavages on the R group, the characteristic ions 158, 133, 116, and 74 were generated (Supplementary Figure 7E). The characteristic ions 126, 115, 97 and 70 were also generated in the MS^3^ spectrum by the cleavage of the fragment 172 (Supplementary Figure 7F). The OH group of *N*^α^ -acetylarginine was lost to produce the characteristic ion *m/z* 200. The fragments *m/z* 175 and 157 were generated by the different cleavages on the R group. The characteristic ions 139, 115 and 70 were produced by the cleavage of amino and carboxyl groups linked with the chiral carbons. The OH group of the *m/z* 200 was lost to produce the *m/z* 183 in the MS^3^ spectrum, and the other fragments were similar to those in the MS^2^ spectrum (Supplementary Figures 7G,H). *N*^2^-(3-Hydroxysuccinoyl)arginine was formed by the substitution of the 3-hydroxysuccinoyl group on the N-terminal of arginine. The typical feature of its cleavage was the loss of 3-hydroxysuccinoyl group to produce the characteristic ion 175 (Supplementary Figure 7I), which was further cleaved to generate the fragments 158, 139, and 60 (Supplementary Figure 7J).

### Identification of some isomers in *Dendrobium officinale*

The accurate molecular weights of the compounds were provided by high-resolution mass spectrometry, which allowed us to deduce the speculative molecular formulas. However, the identification of isomers was less effective. Two pairs of isomers were identified in this study based on their different retention times and distinct multistage fragments. The formula of both L-homophenylalanine and β-phenyl-γ-aminobutyric acid was C_10_H_13_O_2_N, but their retention times were at 6.79 and 12.47 min, respectively (Table 1). The MS^2^ spectra of L-homophenylalanine and β-phenyl-γ-aminobutyric acid are displayed in Supplementary Figures 8A,C, respectively. These mass spectra indicated that the NH_3_ groups were easily lost to form the characteristic ion *m/z* 163. However, the fragment *m/z* 120 produced in the spectrum of β-phenyl-γ-aminobutyric acid was not observed in that of L-homophenylalanine. The MS^3^ spectra presented different fragments, which indicated two different compounds (Supplementary Figures 8B,D). The formula of carbetamide and *N*^δ^ -benzoylornithine were C_12_H_16_O_3_N_2_, while their retention times were at 9.77 and 14.93 min, respectively (Table 1). The MS^2^ and MS^3^ spectra of carbetamide were displayed in Supplementary Figures 8E,F, respectively. The MS^2^ and MS^3^ spectra of *N*^δ^ -benzoylornithine were displayed in Supplementary Figures 8G,H, respectively. These two isomers exhibited different fragmentation patterns. The characteristic ions of carbetamide were the *m/z* 120, 103 and 93 ions, whereas the characteristic ions of *N*^δ^ -benzoylornithine were the *m/z* 219, 205, 175, 158 and 148 ions. The isomerism of the dipeptides resulted from the chirality of the α-carbon and the different positions of peptide bonds between two peptides. In the study, eight dipeptides were tentatively identified. Most of these dipeptides had more than one retention time, suggesting that the isomers existed.

### Identification of the nitrogen-containing glycosides in *Dendrobium officinale*

Compared with the Metlin and Dictionary of Natural Products databases, four nitrogen-containing glycosides were tentatively identified from *D. officinale*. The neutral loss of H_2_O in 5′-*O*-β-D-glucosylpyridoxine was easy to produce the characteristic ion 314. The fragment *m/z* 171 was generated by the cleavage on the O-glucosidic bond and further produced the aglycone 152 by the neutral loss of glucose (Supplementary Figure 9A). The H_2_O on the aglycone of 4-*O*-β-D-glucopyranoside-2,4-benzoxazolediol was easily lost to produce the base peak *m/z* 296. The benzoxazole ion 136 was generated by the cleavage on the O-glucosidic bond (Supplementary Figure 9B). The *m/z* 296 was cleaved to generate the characteristic ions 119 and 94 (Supplementary Figure 9C). The MS^2^ and MS^3^ spectra of *O*-(tri-*O*-acetyl-α-L- rhamnopyranoside)- (4-hydroxybenzyl) methylcarbamic acid were displayed in Supplementary Figures 9D,E, respectively. These mass spectra indicated that the characteristic ion 322 was generated by the cleavage of the ester bond and the side chain. The *m/z* 322 was cleaved to produce the fragments 304 and 160 in the MS^3^ spectrum. Passicapsin was composed of one glucose and a nitrogen-containing aglycone that easily produced the aglycone238 *via* the neutral loss of glucose. The characteristic ions 286 and 148 were cleaved at the O-glucosidic bond between the side chain and the dideoxy glucoside (Supplementary Figure 9F). The fragments 148 and 106 in the MS^3^ spectrum were produced by the cleavage of the aglycone 238 on the O-glucosidic bond. The H_2_O of the aglycone 238 was lost to produce the fragments 220 and 212, and the dideoxy glucoside group was further cleaved to generate the *m/z* 190 and 174 (Supplementary Figure 9G).

## Conclusion

Methyl jasmonate often acts as an elicitor to activate the expression of defense-related genes and the biosynthesis of secondary metabolites. The alkaloids and precursors of *D. officinale* were primarily studied by the SPE-HPLC–LC-MS/MS technique under MeJA treatment. The extraction and purification of the target compounds was optimized, and the suitable conditions of ESI-CID-MS^n^ for the mass spectrometric detection were established. Based on the accurate mass weight with the fragmentation ions provided by the LTQ-Orbitrap mass spectrometer, forty-nine compounds, including amino acids derivatives, dipeptides, nucleotides, guanidines, alkaloids, sphingolipids, and several nitrogen-containing glycosides, were tentatively identified from *D. officinale*. Among them, seven alkaloids were tentatively identified. These findings will serve as a technical guidance for the analyses and drug discoveries of *Dendrobium* alkaloids.

## Data Availability Statement

The original contributions presented in this study are publicly available. This data can be found here: https://doi.org/10.5061/dryad.v6wwpzgzq.

## Author contributions

CS and GL discussed the writing plan and acquired the funding. CS and YZ drafted the manuscript. CS and MM edited the manuscript. All authors have read, reviewed, and approved the submitted version.

## Footnotes

1
https://datadryad.org/stash/share/xacnIUzyyzD69gHfyfz7MWhaKkJ_K4ev7YDd73Oopi4

2
https://metlin.scripps.edu

3
http://dnp.chemnetbase.com

4
http://www.massbank.jp/

## Supplementary Material

Supplementary Figure 1 The mass spectrum of *Dendrobium officinale* extract with the standards. **(A)** The total ion current chromatogram of the methanol extract from *D. officinale* spiked with standards, **(B)** the extracted ion current chromatogram of *m/z* 311.1738, and **(C)** the extracted ion current chromatogram of *m/z* 609.2772.

Supplementary Figure 2 The putative fragmentation pathway of sarpagine. π denotes π-bond dissociation; i denotes inductive cleavage; rH_R_ denotes charge-remote rearrangement; rH_B_ denotes α,β-charge-site rearrangement; rH_C_ denotes γ-charge-site rearrangement; and rH_1,2_ denotes radical-site rearrangement.

Supplementary Figure 3 The putative fragmentation pathway of reserpine. π denotes π-bond dissociation; i denotes inductive cleavage; rH_R_ denotes charge-remote rearrangement; and rH_C_ denotes γ-charge-site rearrangement.

Supplementary Figure 4 The chromatogram and mass spectra of anapheline. **(A)** The extracted ion chromatogram of anapheline, **(B)** the MS spectrum of anapheline, **(C)** the MS^2^ spectrum of anapheline, and **(D)** the MS^3^ spectrum of anapheline.

Supplementary Figure 5 The mass spectra of nucleotides and derivatives. **(A)** The MS^2^ spectrum of 2-hydroxyadenine, **(B)** the MS^3^ spectrum of 2-hydroxyadenine, **(C)** the MS^2^ spectrum of adenosine, **(D)** the MS^3^ spectrum of adenosine, **(E)** the MS^2^ spectrum of guanosine, **(F)** the MS^3^ spectrum of guanosine, **(G)** the MS^2^ spectrum of 1-methyladenosine, **(H)** the MS^3^ spectrum of 1-methyladenosine, **(I)** the MS^2^ spectrum of cytidine, and **(J)** the MS^3^ spectrum of cytidine.

Supplementary Figure 6 The mass spectra of sphingolipids. **(A)** The MS^2^ spectrum of 2-amino-1,3- hexadecanediol, **(B)** the MS^3^ spectrum of 2-amino-1,3- hexadecanediol, **(C)** the MS^2^ spectrum of 2-amino-3-tetradecanol, **(D)** the MS^3^ spectrum of 2-amino-3-tetradecanol, **(E)** the MS^2^ spectrum of 2-amino-1,3,4-octadecanetriol, **(F)** the MS^3^ spectrum of 2-amino-1,3,4-octadecanetriol, **(G)** the MS^2^ spectrum of 2-amino-1,3,4- hexadecanetriol, and **(H)** the MS^3^ spectrum of 2-amino-1,3,4-hexadecanetriol.

Supplementary Figure 7 The mass spectra of amino acids and their derivatives. **(A)** The MS^2^ spectrum of 4-hydroxy-L-threonine, **(B)** the MS^2^ spectrum of urocanic acid, **(C)** the MS^2^ spectrum of *N*^2^-fructopyranosylarginine, **(D)** the MS^3^ spectrum of *N*^2^-fructopyranosylarginine, **(E)** the MS^2^ spectrum of *N*-methylarginine, **(F)** the MS^3^ spectrum of *N*-methylarginine, **(G)** the MS^2^ spectrum of *N*^α^ -acetylarginine, **(H)** the MS^3^ spectrum of *N*^α^ -acetylarginine, **(I)** the MS^2^ spectrum of *N*^2^-(3-hydroxysuccinoyl)arginine, and **(J)** the MS^3^ spectrum of *N*^2^-(3-hydroxysuccinoyl)arginine.

Supplementary Figure 8 The comparative mass spectrum of some isomers. **(A)** The MS^2^ spectrum of L-homophenylalanine, **(B)** the MS^3^ spectrum of L-homophenylalanine, **(C)** the MS^2^ spectrum of β-phenyl-γ-aminobutyric acid, **(D)** the MS^3^ spectrum of β-phenyl-γ-aminobutyric acid, **(E)** the MS^2^ spectrum of carbetamide, **(F)** the MS^3^ spectrum of carbetamide, **(G)** the MS^2^ spectrum of *N*^δ^ -benzoylornithine, and **(H)** the MS^3^ spectrum of *N*^δ^ -benzoylornithine.

Supplementary Figure 9 The mass spectra of nitrogen-containing glycosides. **(A)** The MS^2^ spectrum of 5′-*O*-β-D-glucosylpyridoxine, **(B)** the MS^2^ spectrum of 4-*O*-β-D-glucopyranoside-2,4-benzoxazolediol, **(C)** the MS^3^ spectrum of 4-*O*-β-D-glucopyranoside-2,4-benzoxazolediol, **(D)** the MS^2^ spectrum of *O*-(tri-*O*-acetyl-α-L-rhamnopyranoside)-(4-hydroxy benzyl)methylcarbamic acid, **(E)** the MS^3^ spectrum of *O*-(tri-*O*-acetyl-α-L-rhamnopyranoside)-(4-hydroxy benzyl)methylcarbamic acid, **(F)** the MS^2^ spectrum of passicapsin, and **(G)** the MS^3^ spectrum of passicapsin.
