# Supplementary figures and images for: Identification of alkaloids and related intermediates of Dendrobium officinale by solid-phase extraction coupled with high-performance liquid chromatography tandem mass spectrometry
Source: Front Plant Sci. 2022 Aug 4;13:952051. doi: 10.3389/fpls.2022.952051 (PMC9386266; doi:10.3389/fpls.2022.952051)

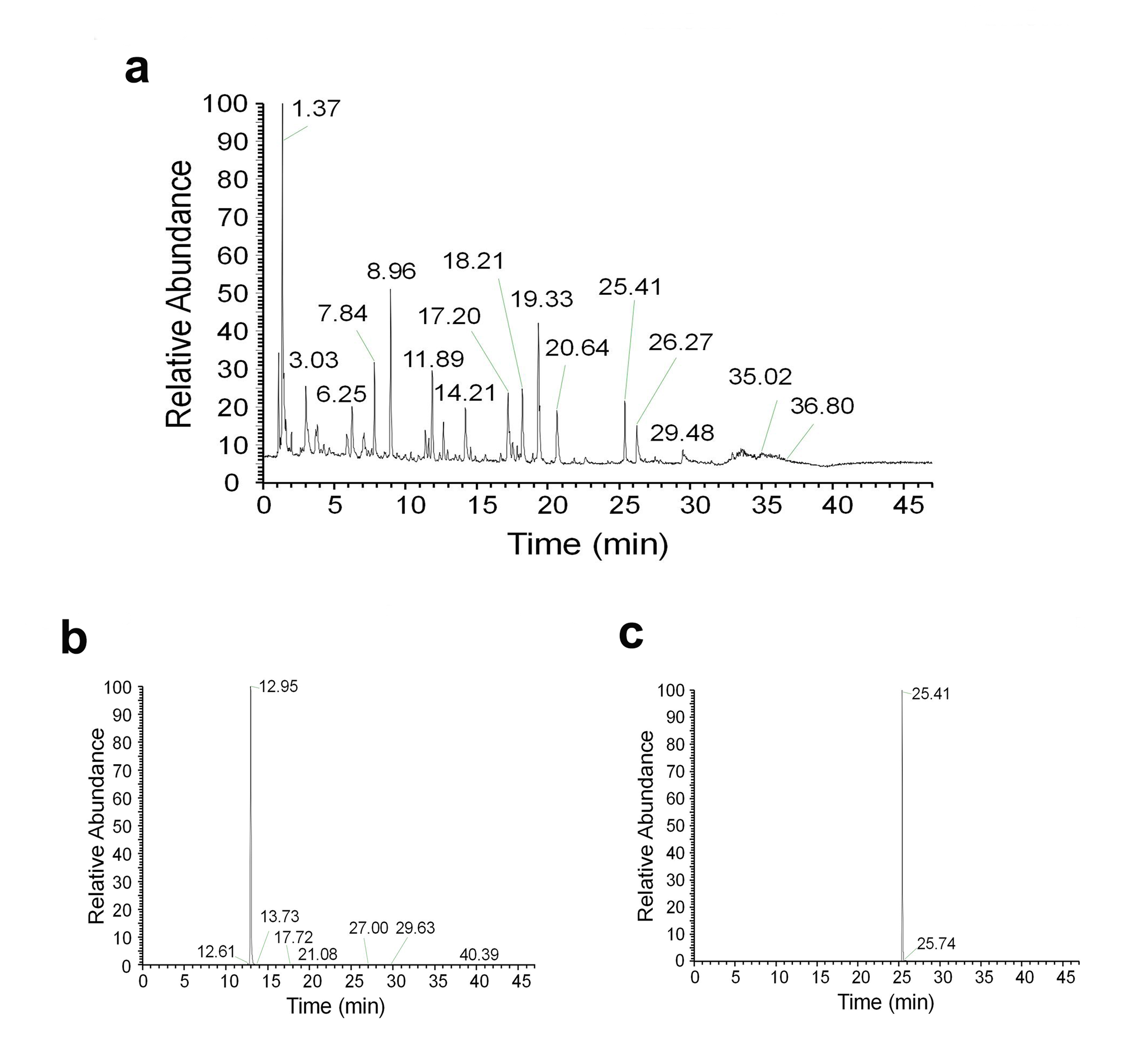

Supplement: Supplementary Figure 1 — The mass spectrum of Dendrobium officinale extract with the standards. (A) The total ion current chromatogram of the methanol extract from D. officinale spiked with standards, (B) the extracted ion current chromatogram of m/z 311.1738, and (C) the extracted ion current chromatogram of m/z 609.2772. [file Image_1.JPEG]

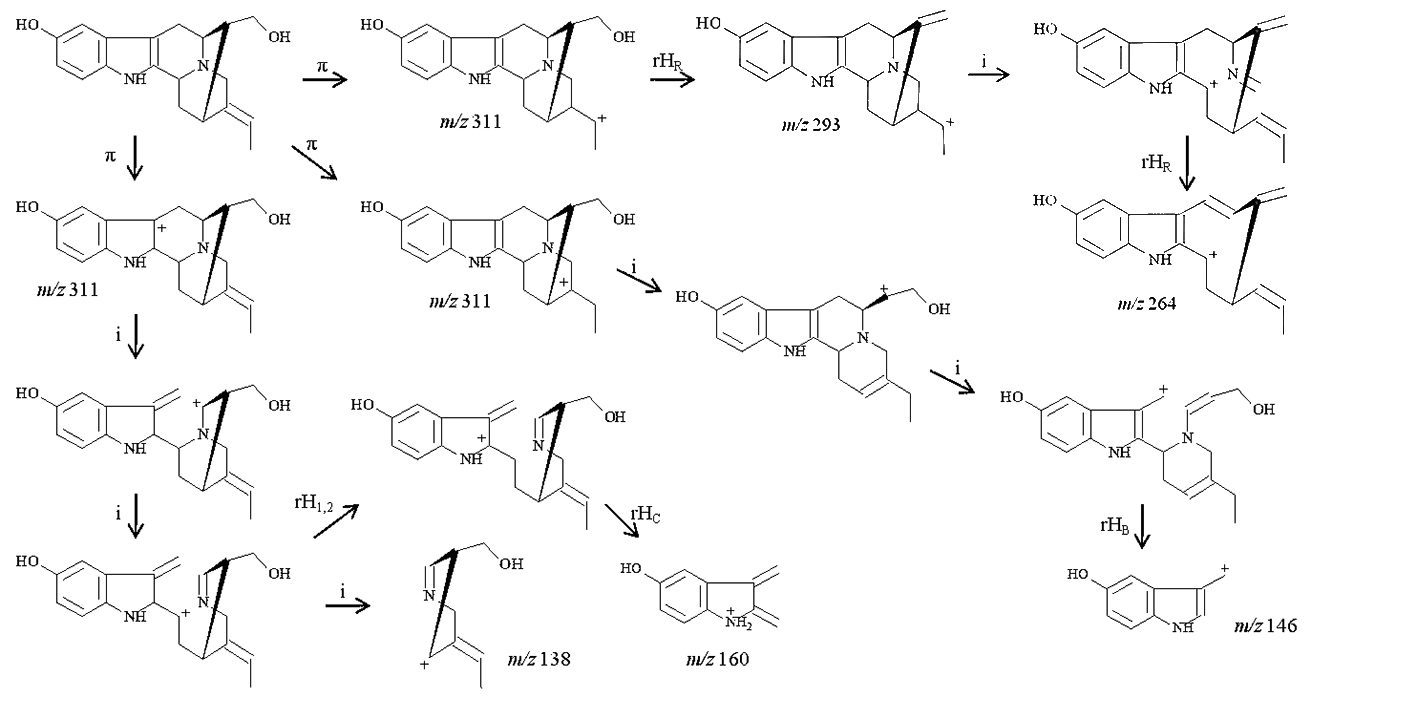

Supplement: Supplementary Figure 2 — The putative fragmentation pathway of sarpagine. π denotes π-bond dissociation; i denotes inductive cleavage; rHR denotes charge-remote rearrangement; rHB denotes α,β-charge-site rearrangement; rHC denotes γ-charge-site rearrangement; and rH1,2 denotes radical-site rearrangement. [file Image_2.JPEG]

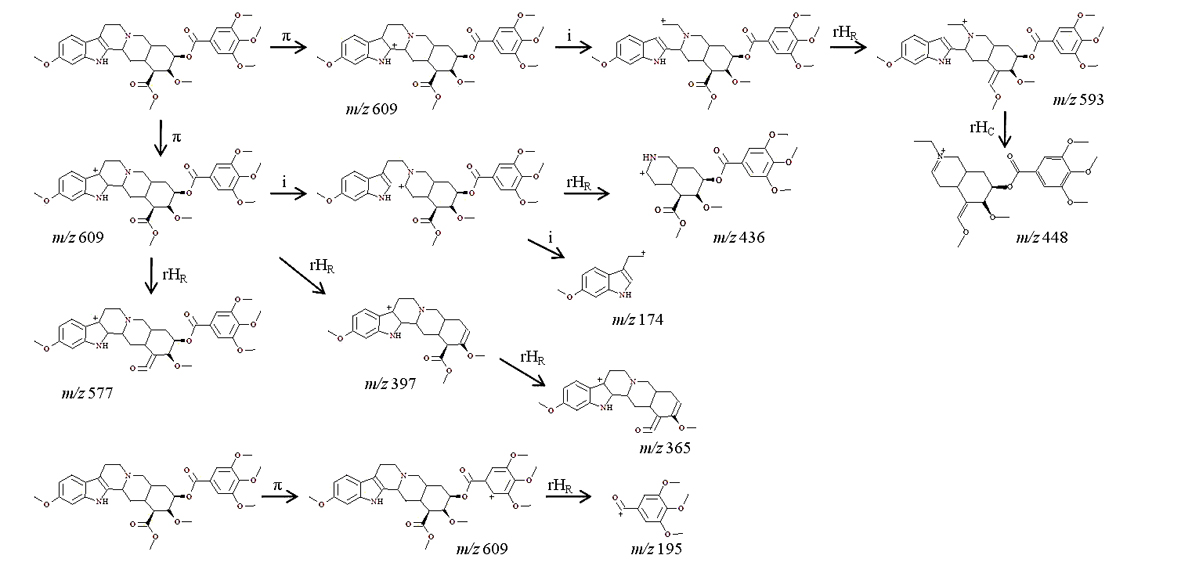

Supplement: Supplementary Figure 3 — The putative fragmentation pathway of reserpine. π denotes π-bond dissociation; i denotes inductive cleavage; rHR denotes charge-remote rearrangement; and rHC denotes γ-charge-site rearrangement. [file Image_3.JPEG]

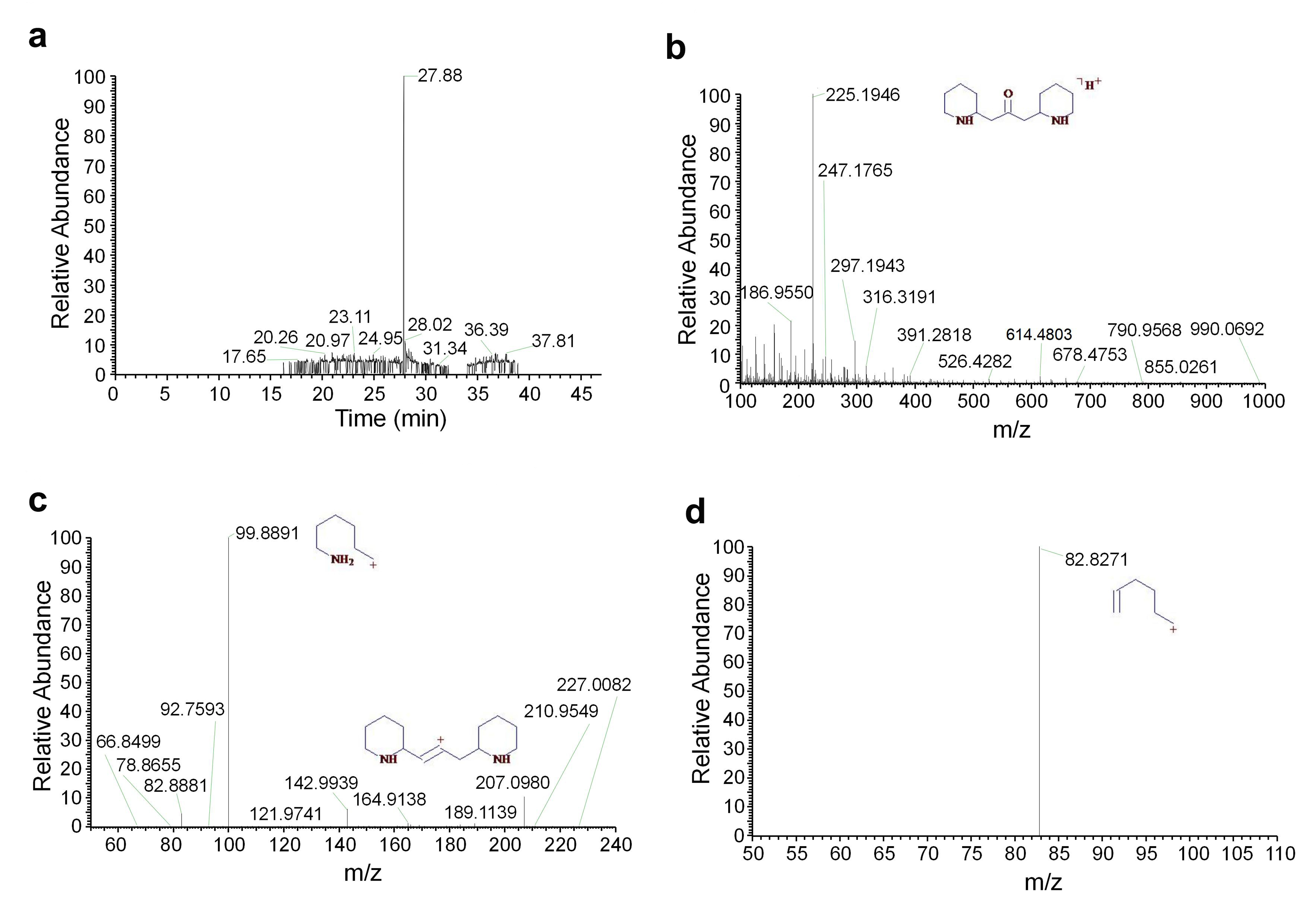

Supplement: Supplementary Figure 4 — The chromatogram and mass spectra of anapheline. (A) The extracted ion chromatogram of anapheline, (B) the MS spectrum of anapheline, (C) the MS2 spectrum of anapheline, and (D) the MS3 spectrum of anapheline. [file Image_4.JPEG]

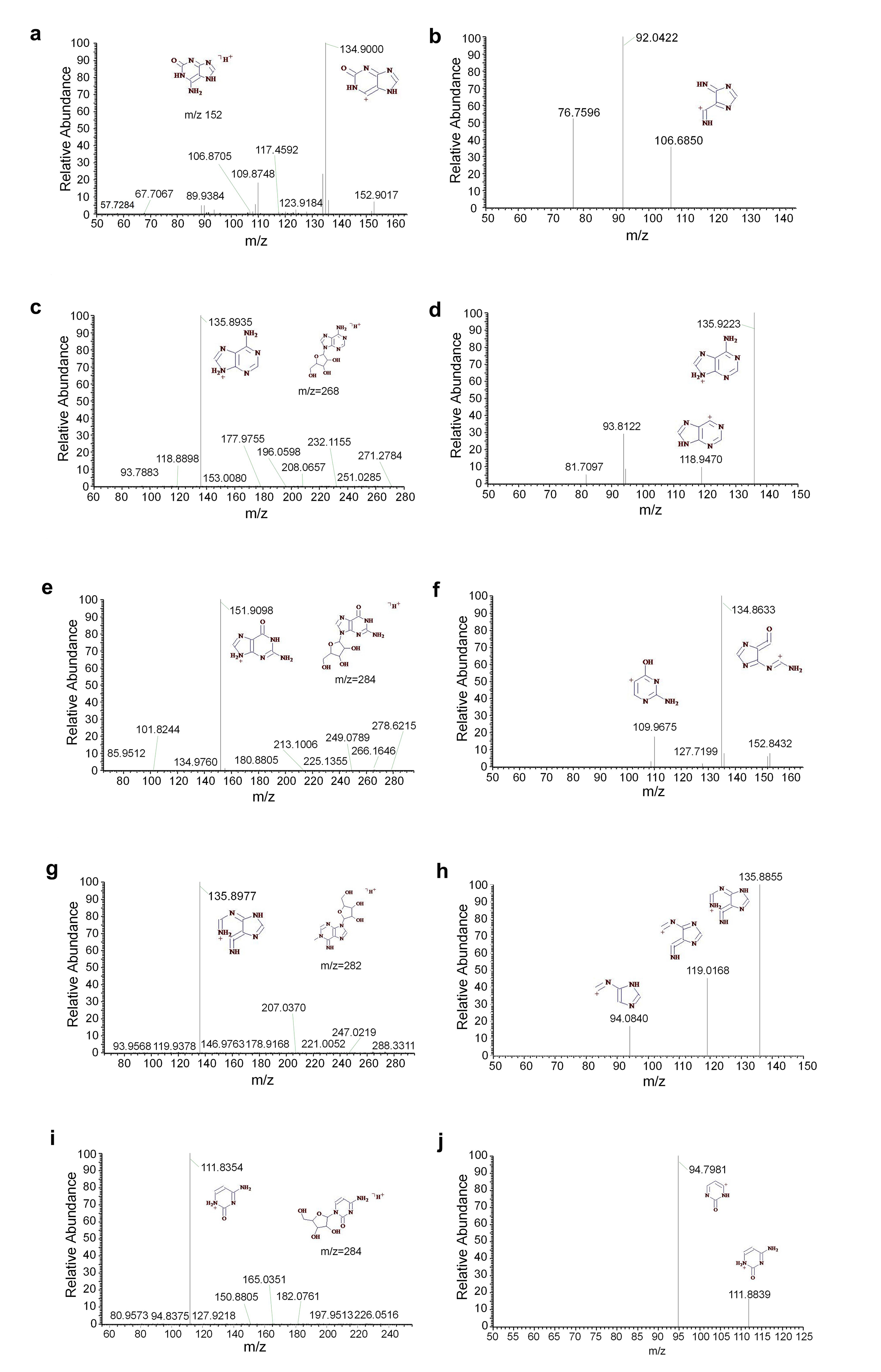

Supplement: Supplementary Figure 5 — The mass spectra of nucleotides and derivatives. (A) The MS2 spectrum of 2-hydroxyadenine, (B) the MS3 spectrum of 2-hydroxyadenine, (C) the MS2 spectrum of adenosine, (D) the MS3 spectrum of adenosine, (E) the MS2 spectrum of guanosine, (F) the MS3 spectrum of guanosine, (G) the MS2 spectrum of 1-methyladenosine, (H) the MS3 spectrum of 1-methyladenosine, (I) the MS2 spectrum of cytidine, and (J) the MS3 spectrum of cytidine. [file Image_5.JPEG]

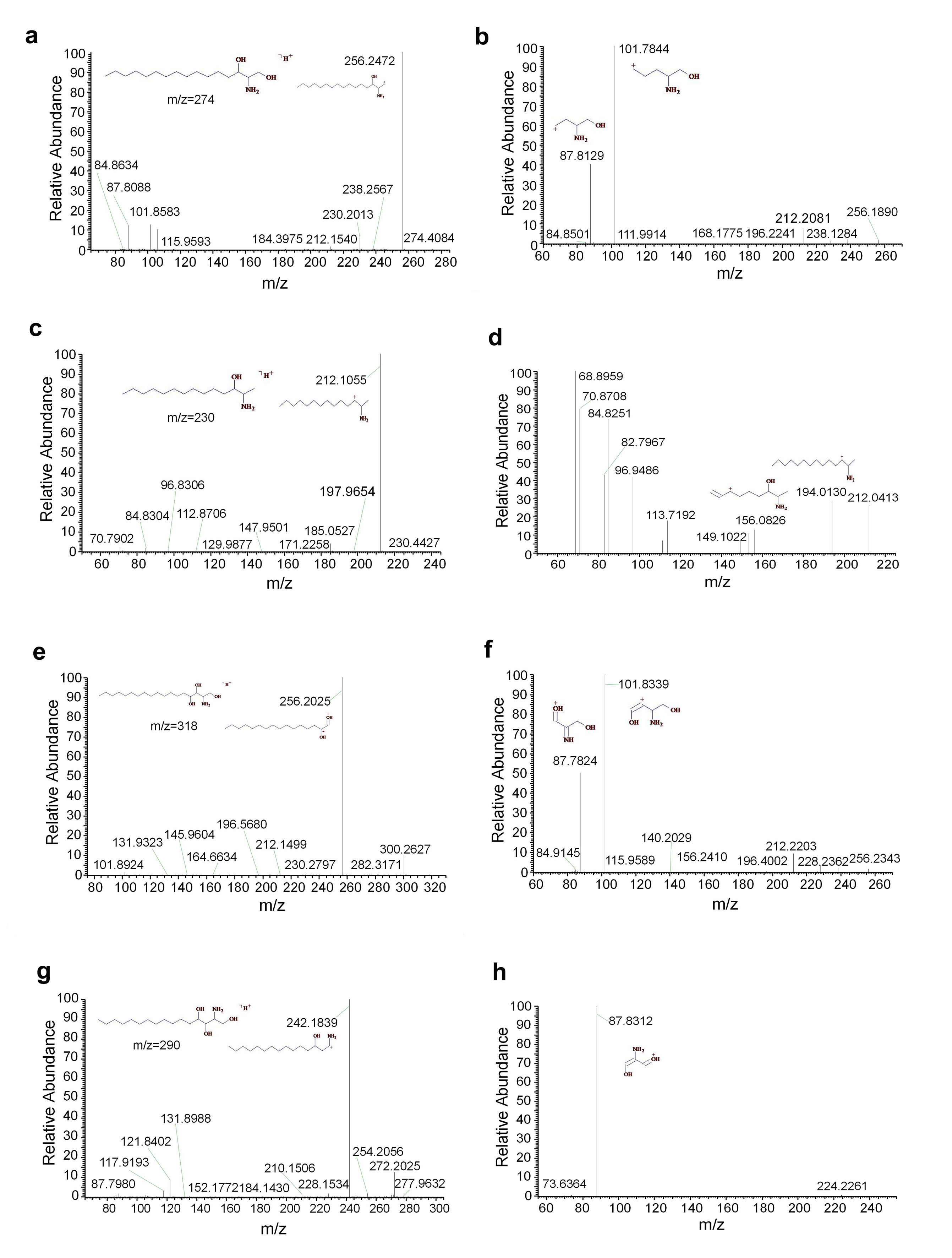

Supplement: Supplementary Figure 6 — The mass spectra of sphingolipids. (A) The MS2 spectrum of 2-amino-1,3- hexadecanediol, (B) the MS3 spectrum of 2-amino-1,3- hexadecanediol, (C) the MS2 spectrum of 2-amino-3-tetradecanol, (D) the MS3 spectrum of 2-amino-3-tetradecanol, (E) the MS2 spectrum of 2-amino-1,3,4-octadecanetriol, (F) the MS3 spectrum of 2-amino-1,3,4-octadecanetriol, (G) the MS2 spectrum of 2-amino-1,3,4- hexadecanetriol, and (H) the MS3 spectrum of 2-amino-1,3,4-hexadecanetriol. [file Image_6.JPEG]

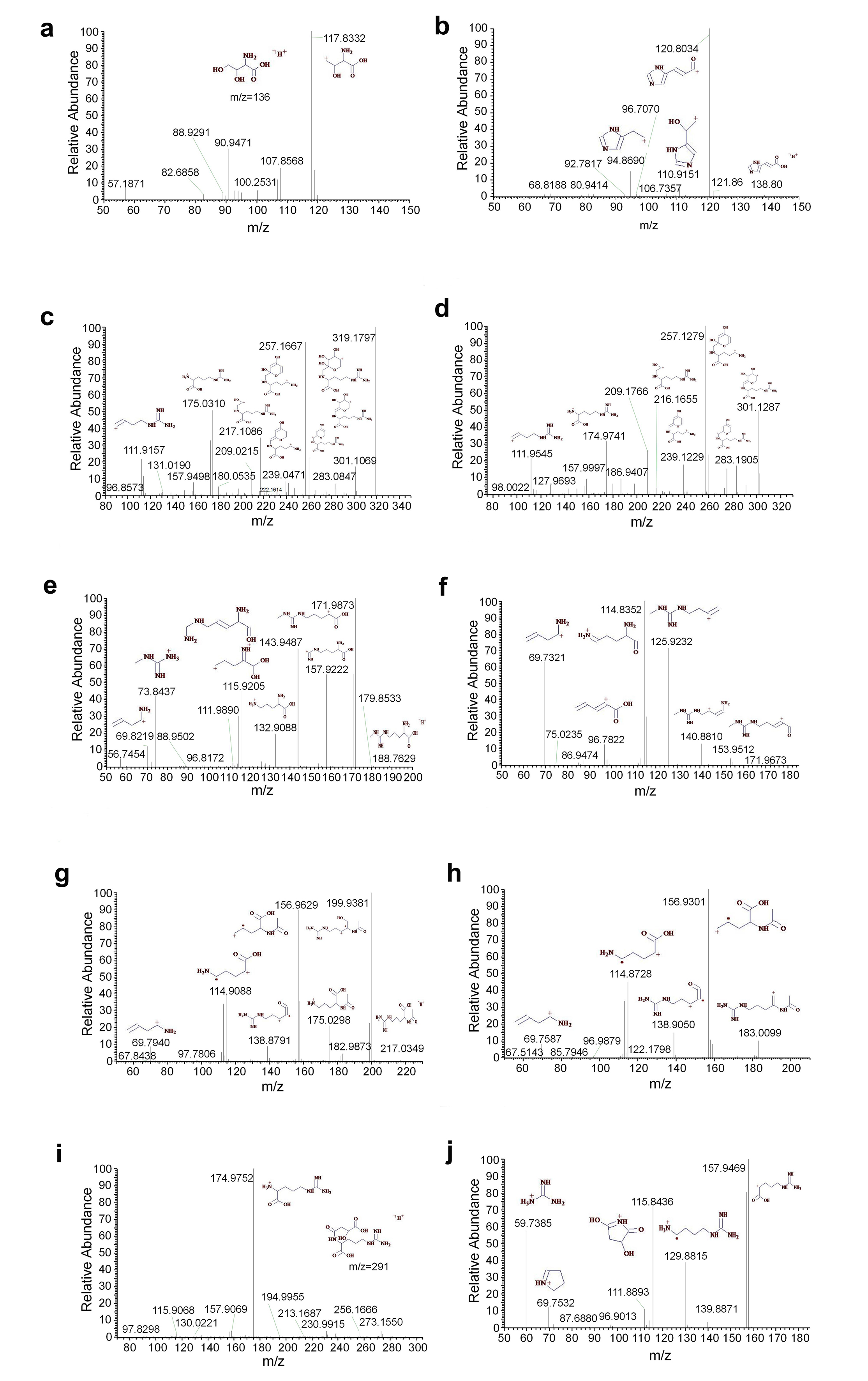

Supplement: Supplementary Figure 7 — The mass spectra of amino acids and their derivatives. (A) The MS2 spectrum of 4-hydroxy-L-threonine, (B) the MS2 spectrum of urocanic acid, (C) the MS2 spectrum of N2-fructopyranosylarginine, (D) the MS3 spectrum of N2-fructopyranosylarginine, (E) the MS2 spectrum of N-methylarginine, (F) the MS3 spectrum of N-methylarginine, (G) the MS2 spectrum of Nα -acetylarginine, (H) the MS3 spectrum of Nα -acetylarginine, (I) the MS2 spectrum of N2-(3-hydroxysuccinoyl)arginine, and (J) the MS3 spectrum of N2-(3-hydroxysuccinoyl)arginine. [file Image_7.JPEG]

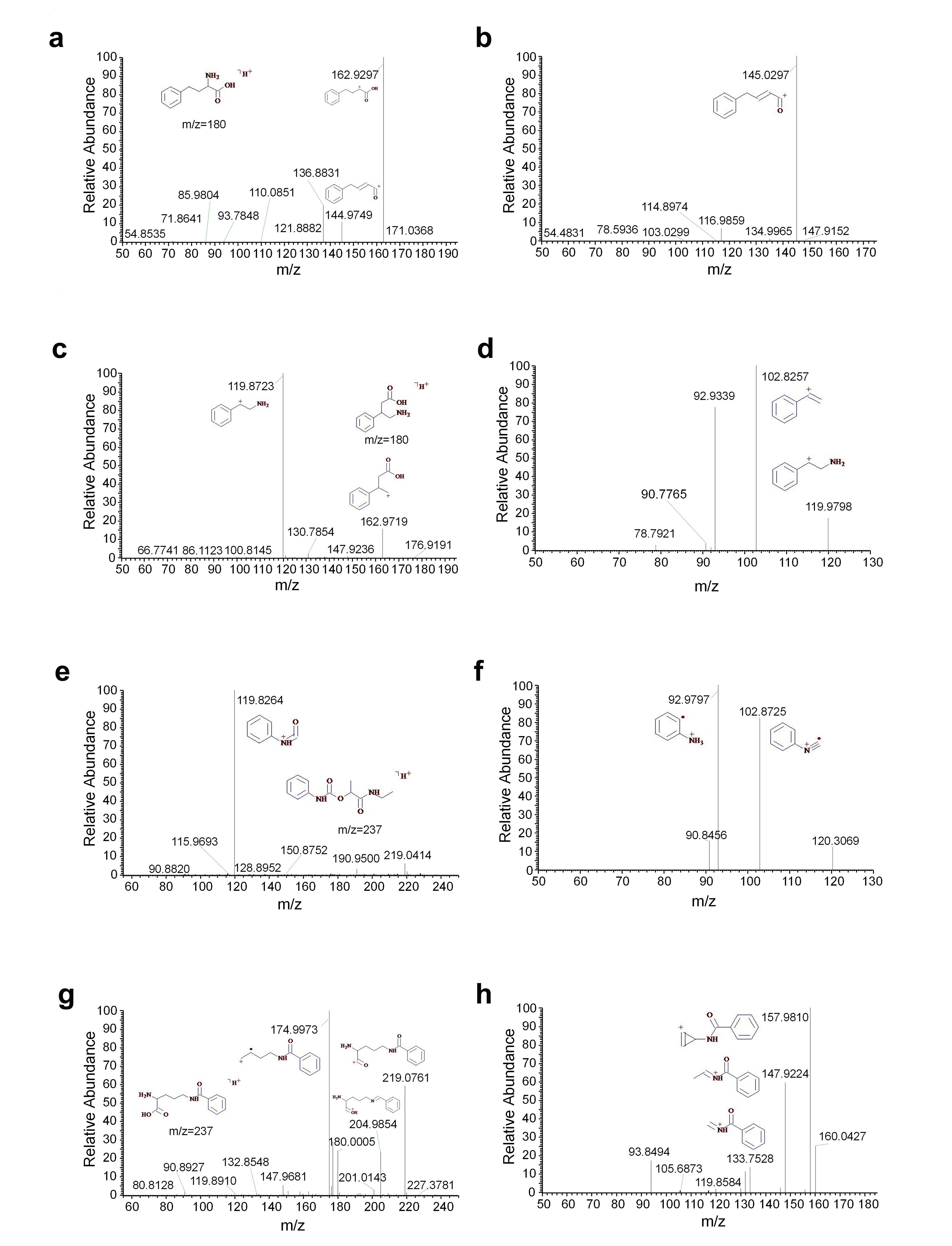

Supplement: Supplementary Figure 8 — The comparative mass spectrum of some isomers. (A) The MS2 spectrum of L-homophenylalanine, (B) the MS3 spectrum of L-homophenylalanine, (C) the MS2 spectrum of β-phenyl-γ-aminobutyric acid, (D) the MS3 spectrum of β-phenyl-γ-aminobutyric acid, (E) the MS2 spectrum of carbetamide, (F) the MS3 spectrum of carbetamide, (G) the MS2 spectrum of Nδ -benzoylornithine, and (H) the MS3 spectrum of Nδ -benzoylornithine. [file Image_8.JPEG]

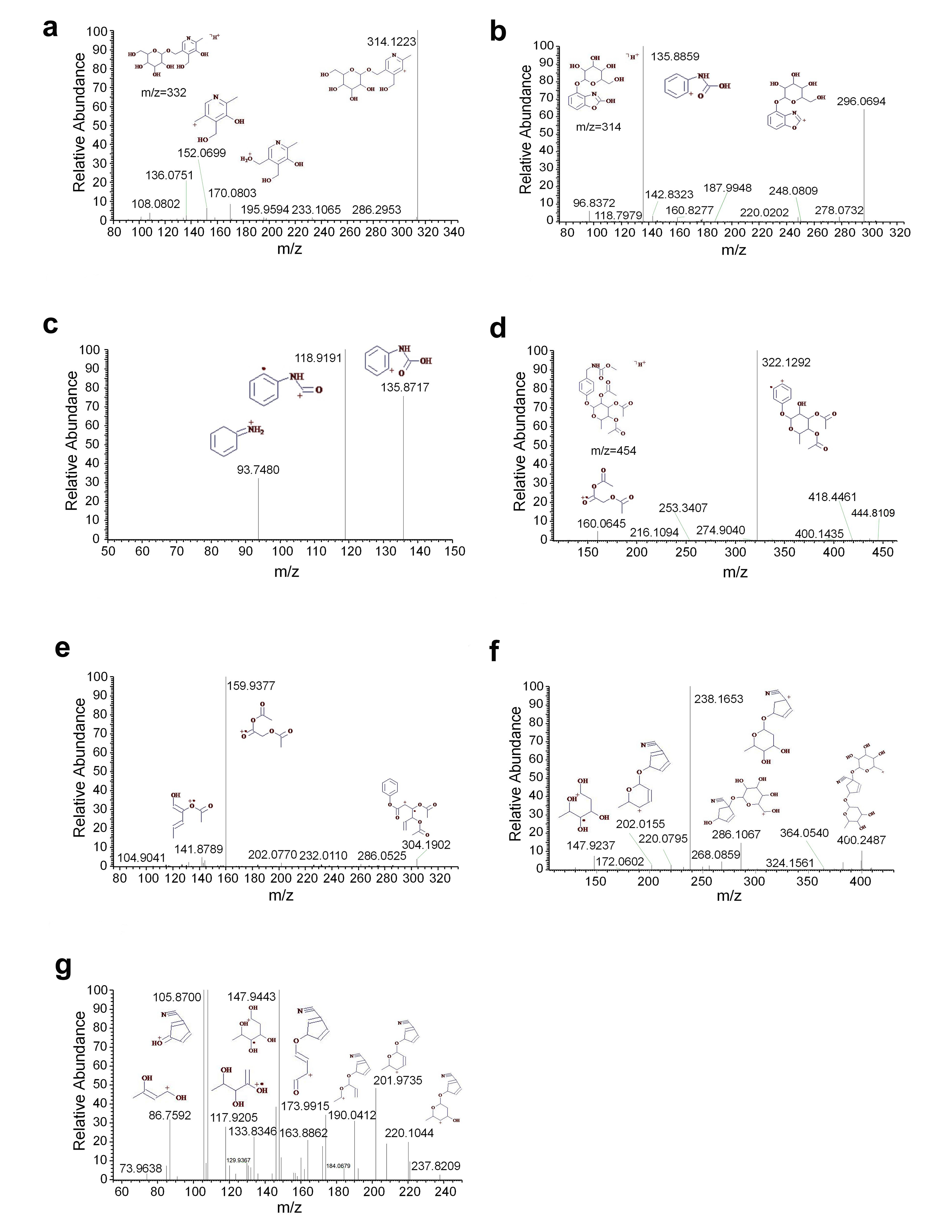

Supplement: Supplementary Figure 9 — The mass spectra of nitrogen-containing glycosides. (A) The MS2 spectrum of 5′-O-β-D-glucosylpyridoxine, (B) the MS2 spectrum of 4-O-β-D-glucopyranoside-2,4-benzoxazolediol, (C) the MS3 spectrum of 4-O-β-D-glucopyranoside-2,4-benzoxazolediol, (D) the MS2 spectrum of O-(tri-O-acetyl-α-L-rhamnopyranoside)-(4-hydroxy benzyl)methylcarbamic acid, (E) the MS3 spectrum of O-(tri-O-acetyl-α-L-rhamnopyranoside)-(4-hydroxy benzyl)methylcarbamic acid, (F) the MS2 spectrum of passicapsin, and (G) the MS3 spectrum of passicapsin. [file Image_9.JPEG]
